# Peanut (*Arachis hypogaea*) sprout prevents high-fat diet-induced cognitive impairment by improving mitochondrial function

**DOI:** 10.1038/s41598-022-10520-5

**Published:** 2022-04-13

**Authors:** Seon Kyeong Park, Hyo Lim Lee, Jin Yong Kang, Jong Min Kim, Ho Jin Heo

**Affiliations:** 1 Division of Applied Life Science, Institute of Agriculture and Life Science (BK21), Gyeongsang National University, Jinju, 52828 Republic of Korea; 2 Korea Food Research Institute, Wanju-gun, 55365 Republic of Korea; 3 Advanced Process Technology and Fermentation Research Group, World Institute of Kimchi, Gwangju, 61755 Republic of Korea

**Keywords:** Biochemistry, Neuroscience, Diseases

## Abstract

This study was performed to evaluate the improvement effect of the ethyl acetate fraction from peanut (*Arachis hypogaea*) sprout (EFPS) on high-fat diet (HFD)-induced cognitive deficits in C57BL/6 mice. Mice were randomly divided four groups (n = 13) as control (normal chow), HFD, EFPS 20 (20 mg/kg of body weight; intragastric administration) and EFPS 50 (50 mg/kg of body weight; intragastric administration) groups. HFD was provide for 15 weeks excepting control group. EFPS ameliorated cognitive dysfunction in Y-maze, passive avoidance test and Morris water maze test. EFPS significantly improved glucose tolerance and serum lipid profile, and reduced body weight. EFPS ameliorated oxidative stress by regulating MDA levels and SOD activity in liver and brain tissues. In addition, EFPS restored brain mitochondrial dysfunction related to energy metabolism. Moreover, the bioactive compounds of EFPS were identified as di-caffeic acid, caffeic acid, dihydrokaempferol-hexoside, di-*p*-coumaroyl tartaric acid isomer and group B soyasaponins using ultra-performance liquid chromatography-quadrupole-time-of-flight (UPLC-Q-TOF) mass spectrometry. These results show that EFPS can improve cognitive functions in HFD-induced diabetic mice.

## Introduction

Metabolic syndrome is a prevalent public health problem characterized by the concurrence of several metabolic dysfunctions including diabetes, hyperglycemia, obesity, impaired glucose tolerance and dyslipidemia^1^. Type 2 diabetes mellitus (T2DM) is a well-known cause of metabolic syndrome related to hyperglycemia and insulin secretion disorders^2^. In particular, excessive accumulation of fat derived from obesity is known to be an important risk factor for T2DM^3^. Sustained hyperglycemia caused by T2DM is known to cause injury to various organs by promoting the production of reactive oxygen species (ROS)^4^. In particular, excessive production of ROS damages cerebral neurons, leading to cognitive impairment. Likewise, ROS can cause systemic inflammation through complex processes, and cause brain mitochondrial dysfunction^5^. Mitochondria play an important role in maintaining cell homeostasis by producing more than 90% of its energy through oxidative phosphorylation, but damaged mitochondria continuously induce oxidative stress and apoptosis^6^. As a result, hyperglycemia from obesity contributes to the pathogenesis of insulin resistance, a key feature of T2DM, which may lead to the development of neuronal dysfunction in Alzheimer’s disease (AD)^7^.

Plant-derived natural resources are widely used as preventive and alternative therapies for various diseases, including metabolic syndrome^8^. For example, natural phenolic compounds have been reported to be effective in diabetes and neurodegenerative diseases in various studies^9,10^. Peanut (*Arachis hypogaea*) sprouts are rich in various polyphenols such as *p*-coumaric acid and trans-ferulic acid^11^. Peanut sprouts contain a variety of bioactive compounds that have various physiological functions, such as anti-inflammation, anti-oxidation and anti-obesity activity^12–14^. However, studies on the effects of peanut sprouts on cognitive dysfunction induced by a high-fat diet are insufficient. Therefore, this study was conducted to confirm the cognitive dysfunction improvement effect of ethyl acetate fraction from peanut sprout (EFPS) in high-fat diet (HFD)-induced diabetic mice.

## Results

### Body and organ weight

HFD significantly increased body weight, including liver, spleen, kidney, testis and epididymal fat weights (Table 1). The body weight of the HFD group (49.00 ± 1.69 g) increased more than the control (31.88 ± 1.89 g) at 15 weeks. However, the body weight of the EFPS 20 (HFD + 20 mg/kg of body weight/day of EFPS) (47.13 ± 1.89 g) and EFPS 50 (HFD + 50 mg/kg of body weight/day of EFPS) (46.00 ± 3.16 g) groups statistically decreased more than the HFD group.

**Table 1:** Effect of ethyl acetate fractions from peanut (*Arachis hypogaea*) sprout (EFPS) on organ weight change (Unit: g).

	Body weight	Liver	Spleen	Kidney	Testis	Epididymal fat
Control	31.88 ± 1.89^c^	1.16 ± 0.06^d^	0.07 ± 0.01^c^	0.32 ± 0.01^c^	0.19 ± 0.01^bc^	0.92 ± 0.21^c^
HFD	49.00 ± 1.69^a^	2.73 ± 0.11^a^	0.12 ± 0.01^a^	0.44 ± 0.01^a^	0.23 ± 0.00^a^	1.58 ± 0.06^a^
EFPS 20	47.13 ± 1.89^ab^	2.05 ± 0.28^b^	0.08 ± 0.01^bc^	0.38 ± 0.01^b^	0.20 ± 0.01^b^	1.32 ± 0.14^b^
EFPS 50	46.00 ± 3.16^b^	1.39 ± 0.17^c^	0.08 ± 0.01^b^	0.36 ± 0.01^b^	0.19 ± 0.02^c^	1.34 ± 0.13^b^

The results were shown with mean ± SD (*n* = 5).

Data were statistically considered at *p* < 0.05, and different small letters represented a statistical difference.

### Fasting blood glucose and IPGTT

**Figure 1:**
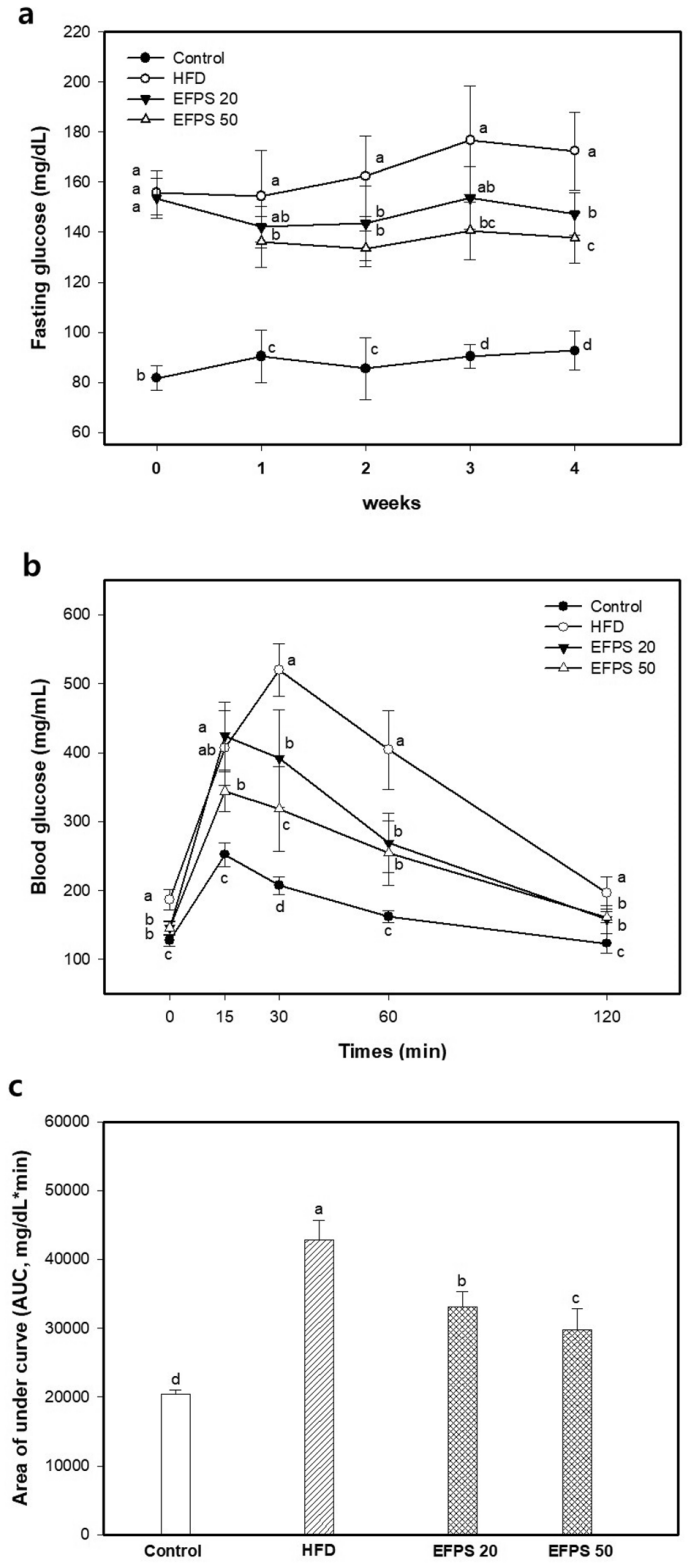
Effect of ethyl acetate fractions from peanut (*Arachis hypogaea*) sprout (EFPS) on fasting glucose level (**a**) and intraperitoneal blood glucose tolerance test (IPGTT) (**b**) in high-fat diet-induced diabetic mice and area under the curve (AUC) (**c**) in IPGTT. Results shown are mean ± SD (*n* = 5). Data were statistically considered at *p* < 0.05, and different small letters represent statistical difference.

Fasting blood glucose and IPGTT results are shown in Fig. 1. Before EFPS ingestion, the HFD group (159.68 ± 41.21 mg/dL) presented a significantly increased fasting blood glucose compared to the control group (92.62 ± 29.36 mg/dL), but for the diet period, the fasting blood glucose of the EFPS groups were significantly decreased compared to the HFD group (Fig. 1a). Fasting blood glucose level at 4 weeks in EFPS groups (EFPS20; 147.25 ± 8.53 mg/dL and EFPS50; 137.63 ± 9.91 mg/dL) were significantly more decreased than HFD group (172.37 ± 15.67 mg/dL). Also, in the IPGTT results, it was consistently measured that glucose tolerance was improved in the EFPS groups compared to the HFD group (Fig. 1b). The results of AUC in EFPS groups (EFPS 20; 33,092.81 ± 2,247.64 mg/dL and EFPS 50; 29,785.31 ± 3,102.99 mg/dL) were reduced than the HFD group (42,840.00 ± 2,849.98 mg/dL) (Fig. 1c).

### Behavioral tests

**Figure 2:**
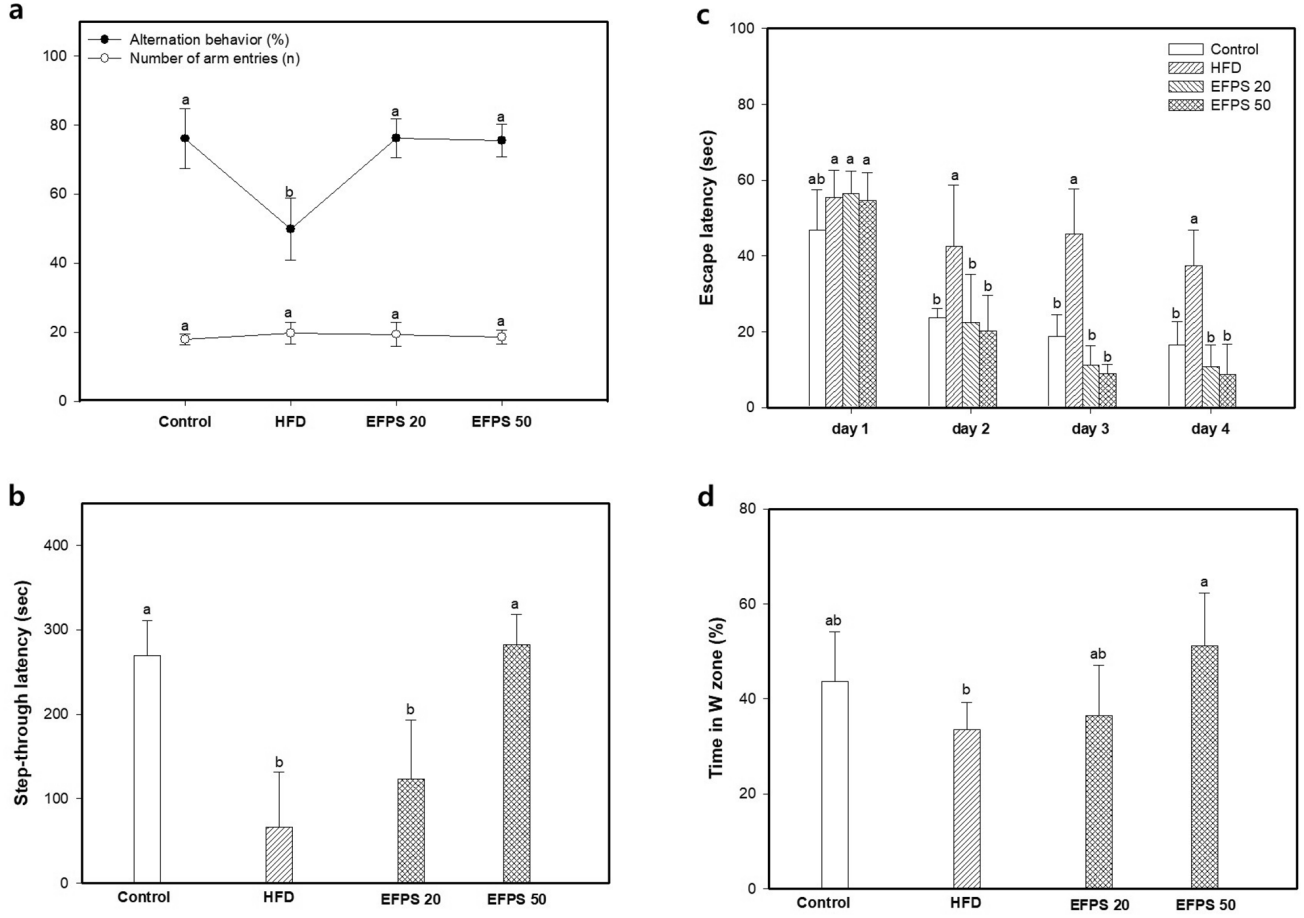
Effect of ethyl acetate fractions from peanut (*Arachis hypogaea*) sprout (EFPS) in high-fat diet-induced diabetic mice on alternation behavior and number of arm entries (**a**) in Y-maze test, step-through latency (**b**) in passive avoidance test, escape latency (**c**) of hidden platform test, W zone crossings of probe test (**d**) in Morris water maze test. Results shown are mean ± SD (*n* = 5). Data were statistically considered at *p* < 0.05, and different small letters represent statistical difference.

Y-maze, passive avoidance, and MWM tests were performed to estimate the effect of EFPS on cognitive function. In the result of Y-maze test, the number of arm items was no significant difference between all groups (Fig. 2a). The alternation behavior of the HFD group (49.85%) decreased compared with the control group (76.03 ± 8.59%). However, the EFPS groups (EFPS 20; 76.16 ± 5.62% and EFPS 50; 75.47 ± 4.67%) effectively improved alternation behavior compared with the HFD group.

In the results of passive avoidance test, the HFD group had lower step-through latency (66.67 ± 65.26 s) in retention than the control groups (Fig. 2b). However, the EFPS-treated groups had higher step-through latency (EFPS 20; 123.75 ± 69.45 s and EFPS 50; 282.00 ± 36.00 s) in retention compared to the HFD group.

The MWM test was performed to evaluated spatial learning and long-term memory function, and the results are shown in Fig. 2c–d. There were differences in escape latency among each of the groups on the first day. The latency in finding the waterlogged platform of all groups decreased every day, but HFD group was significantly longer than the other groups. Case of probe test, the HFD group showed a 33.60 ± 5.67% decrease in the time spent in the quadrant with the platform (Fig. 2d). Compared to the HFD group, the time spent in the quadrant with the platform of the EFPS 50 group increased significantly by 51.17 ± 11.20%.

### Serum biochemicals

The levels of serum biochemicals are shown in Tables 2 and 3. The levels of TCHO, TG and LDLC were remarkably high in the HFD group (236.89 ± 33.39 mg/dL, 154.58 ± 17.72 mg/dL and 39.44 ± 2.61 mg/dL) compared to the control group (110.20 ± 7.73 mg/dL, 109.60 ± 11.24 mg/dL and 16.28 ± 6.11 mg/dL) (Table 2). However, these biomarkers levels decreased in the EFPS groups (EFPS20; 198.00 ± 47.63 mg/dL, 137.50 ± 18.64 mg/dL, and 29.96 ± 6.57 mg/dL, EFPS 50; 198.75 ± 17.33 mg/dL, 126.00 ± 28.19 mg/dL and 22.84 ± 2.96 mg/dL). Additionally, HTR (%) showed higher levels in the EFPS groups (EFPS 20; 70.36 ± 6.67% and EFPS 50; 75.03 ± 5.56%) compared to the HFD group (66.49 ± 9.27%).

**Table 2:** Effect of ethyl acetate fraction from peanut (*Arachis hypogaea*) sprout (EFPS) on total cholesterol (TCHO), triglyceride (TG), low density lipoprotein cholesterol (LDLC), high density lipoprotein cholesterol and TCHO ratio (HTR) in serum.

	TCHO (mg/dL)	TG (mg/dL)	LDLC (mg/dL)	HTR (%)
Control	110.20 ± 7.73^c^	109.60 ± 11.24^c^	16.28 ± 6.11^d^	63.05 ± 2.74^c^
HFD	236.89 ± 33.39^a^	154.58 ± 17.72^a^	39.44 ± 2.61^a^	66.49 ± 9.27^bc^
EFPS 20	198.00 ± 47.63^b^	137.50 ± 18.64^ab^	29.96 ± 6.57^b^	70.36 ± 6.67^ab^
EFPS 50	198.75 ± 17.33^b^	126.00 ± 28.19^bc^	22.84 ± 2.96^c^	75.03 ± 5.56^a^

The results were shown with mean ± SD (*n* = 5).

Data were statistically considered at *p* < 0.05, and different small letters represented a statistical difference.

**Table 3:** Effect of ethyl acetate fraction from peanut (*Arachis hypogaea*) sprout (EFPS) on glutamic oxaloacetic transaminase (GOT), glutamine pyruvic transaminase (GPT), lactate dehydrogenase (LDH), blood urea nitrogen (BUN) and creatine (CRE) in serum.

	GOT (U/L)	GPT (U/L)	LDH (U/L)	BUN (mg/dL)	CRE (mg/dL)
Control	50.40 ± 1.52^d^	33.60 ± 3.05^d^	265.80 ± 67.86^c^	13.70 ± 1.43^b^	0.12 ± 0.04^a^
HFD	169.20 ± 19.40^a^	243.40 ± 36.18^a^	722.14 ± 72.66^a^	15.94 ± 1.10^a^	0.16 ± 0.05^a^
EFPS 20	111.00 ± 7.78^b^	151.20 ± 33.39^b^	427.60 ± 62.28^b^	14.54 ± 0.62^b^	0.14 ± 0.05^a^
EFPS 50	90.40 ± 6.11^c^	98.40 ± 12.42^c^	429.20 ± 67.75^b^	14.58 ± 1.08^b^	0.14 ± 0.05^a^

The results were shown with mean ± SD (*n* = 5).

Data were statistically considered at *p* < 0.05, and different small letters represented a statistical difference.

Hepatic and renal toxicity was confirmed by serum GOT, GPT, BUN, CRE and LDH levels (Table 3). HFD consumption (169.20 ± 19.40 U/L, 243.40 ± 36.18 U/L, 15.94 ± 1.10 mg/dL and 722.14 ± 72.66 U/L) caused an increase in GOT, GTP, LDH, and BUN levels compared to the control group (50.40 ± 1.52 U/L, 33.60 ± 3.05 U/L, 265.80 ± 67.86 U/L and 13.70 ± 1.43 mg/dL). EFPS treatment (EFPS 20; 111.00 ± 7.78 U/L, 151.20 ± 33.39 U/L, 427.20 ± 62.28 U/L and 14.54 ± 0.62 mg/ dL, EFPS 50; 90.40 ± 6.11 U/L, 98.40 ± 12.42 U/L, 429.20 ± 67.75 U/L and 14.58 ± 1.08 mg/dL) lowered the level of GOT, GTP, BUN and LDH compared to the HFD group. There was no significant difference in serum CRE level between all groups.

### Antioxidant effect

**Figure 3:**
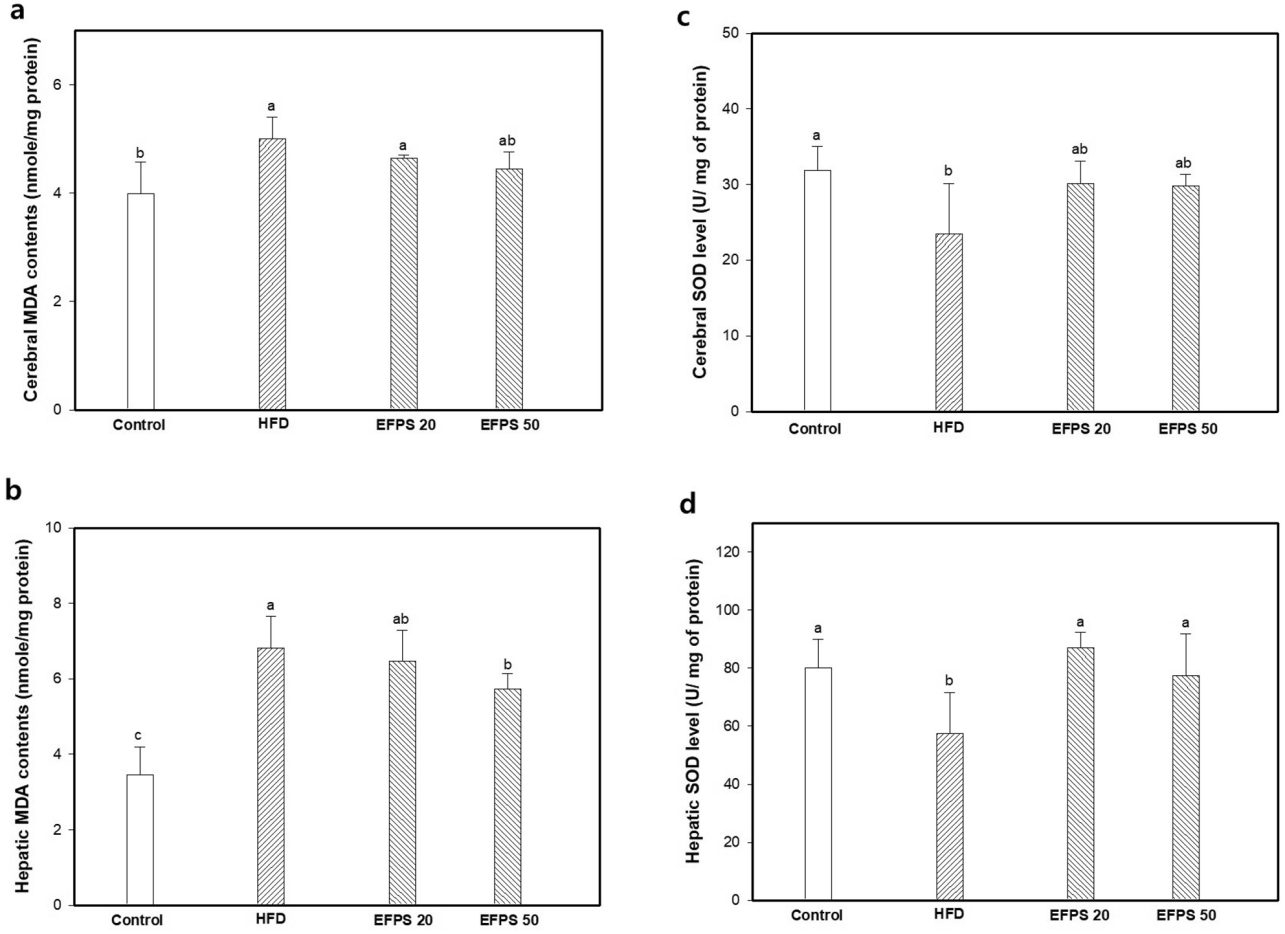
Effect of ethyl acetate fractions from peanut (*Arachis hypogaea*) sprout (EFPS) of malondialdehyde (MDA) contents (**a**,**b**) and superoxide dismutase (SOD) level (**c**,**d**) from brain and liver homogenates of high fat-induced diabetic mice. Results shown are means ± SD (n = 5). Data were statistically considered at *p* < 0.05, and different small letters represent statistical differences.

The protective effect of EFPS against oxidative stress in liver and brain tissue was confirmed by measuring MDA contents and SOD level (Fig. 3). The MDA contents in the brain (4.50 ± 0.40 nmole/mg of protein) and liver (6.81 ± 0.85 nmole/mg of protein) tissues of the HFD group increased compared to the control group (3.99 ± 0.58 and 3.45 ± 0.76 nmole/mg of protein) (Fig. 3a,b). However, the hepatic MDA contents showed decreased in the EFPS 50 group (cerebral tissue; 4.45 ± 0.31 and hepatic tissue; 5.72 ± 0.41 nmole/mg of protein) compared to the HFD group. There is no significant difference between EFPS 50 group and HFD group on cerebral MDA contents.

Brain and liver SOD level are presented in Fig. 3c,d. The SOD level in the cerebral (23.49 ± 6.69 U/mg of protein) and hepatic (57.49 ± 14.10 U/mg of protein) tissues of the HFD group decreased compared to the control group (31.83 ± 3.20 and 80.15 ± 9.67 U/mg of protein). However, the SOD level increased in the EFPS groups (EFPS 20; 86.92 ± 5.42 U/mg of protein, EFPS 50; 77.37 ± 14.48 U/mg of protein) by decreasing hepatic oxidative stress compared to the HFD group. Cerebral SOD level in EFPS groups showed no significant difference compared to HFD group.

### Mitochondrial parameters

**Figure 4:**
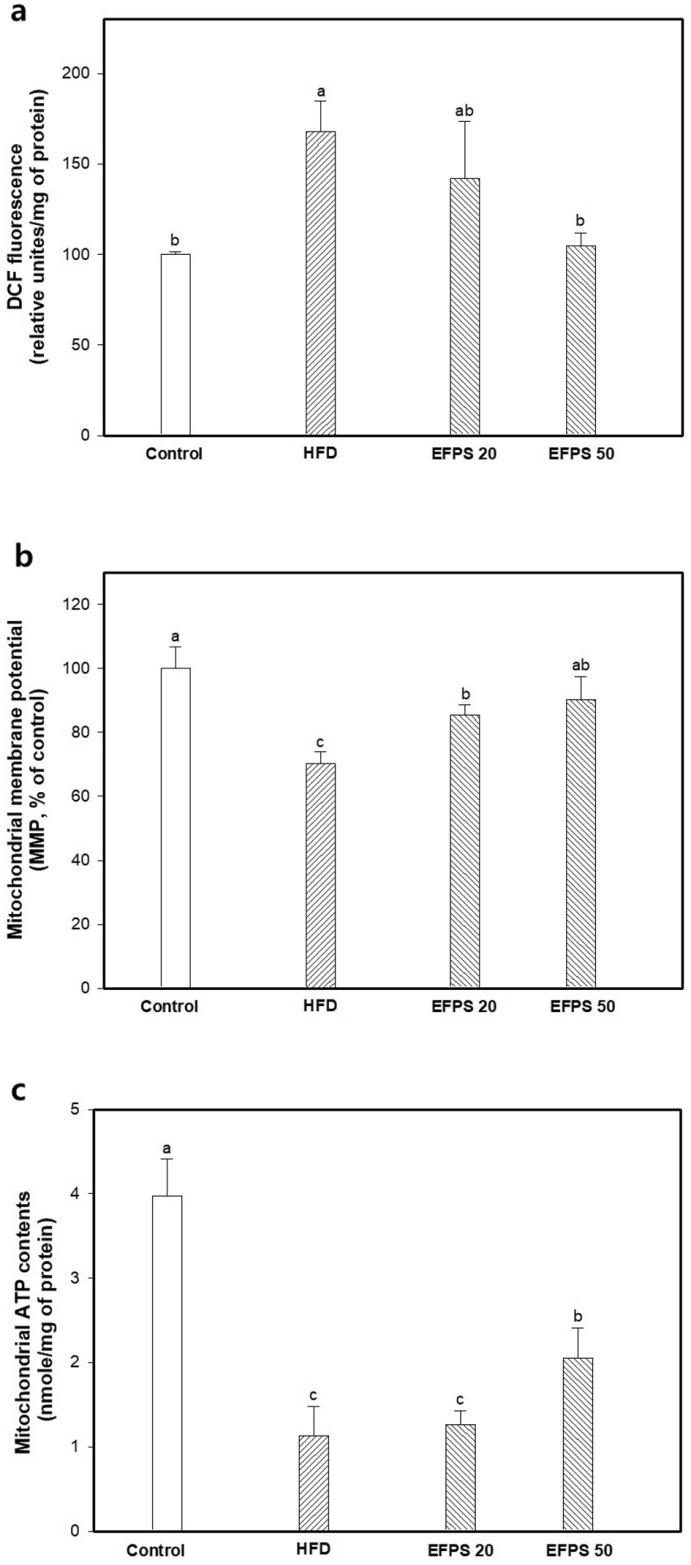
Effect of ethyl acetate fractions from peanut (*Arachis hypogaea*) sprout (EFPS) on mitochondrial ROS production (**a**), mitochondrial membrane potential (MMP) (**b**) and mitochondrial ATP (**c**). Results shown are means ± SD (n = 5). Data were statistically considered at *p* < 0.05, and different small letters represent statistical differences.

The effect of EFPS on brain mitochondrial dysfunction was assessed by measuring ROS production, MMP and ATP contents (Fig. 4). The mitochondria ROS production was measured on using DCF-DA (Fig. 4a). The ROS production in the HFD group increased fluorescence intensity (167.71 ± 16.83%) more than the control group (100.00 ± 1.52%). Treatment with EFPS 50 (104.63 ± 7.26%) reduced mitochondrial ROS content more than treatment with EFPS 20 (141.97 ± 31.55%). The result of MMP (Fig. 4b), it was decreased in the HFD group (70.25 ± 3.60%) compared to the control group (100.00 ± 6.63%). However, MMP was improved in the EFPS groups (EFPS 20; 85.36 ± 3.39% and EFPS 50; 90.33 ± 7.10%) compared to the HFD group. Mitochondrial ATP content more decreased in the HFD group (1.13 ± 0.35 nmole/mg of protein) than control group (3.97 ± 0.44 nmole/mg of protein). Treatment with EFPS 50 (2.05 ± 0.37 nmole/mg of protein) improved the decrease in ATP production compared to the HFD group.

### Neuronal apoptosis

**Figure 5:**
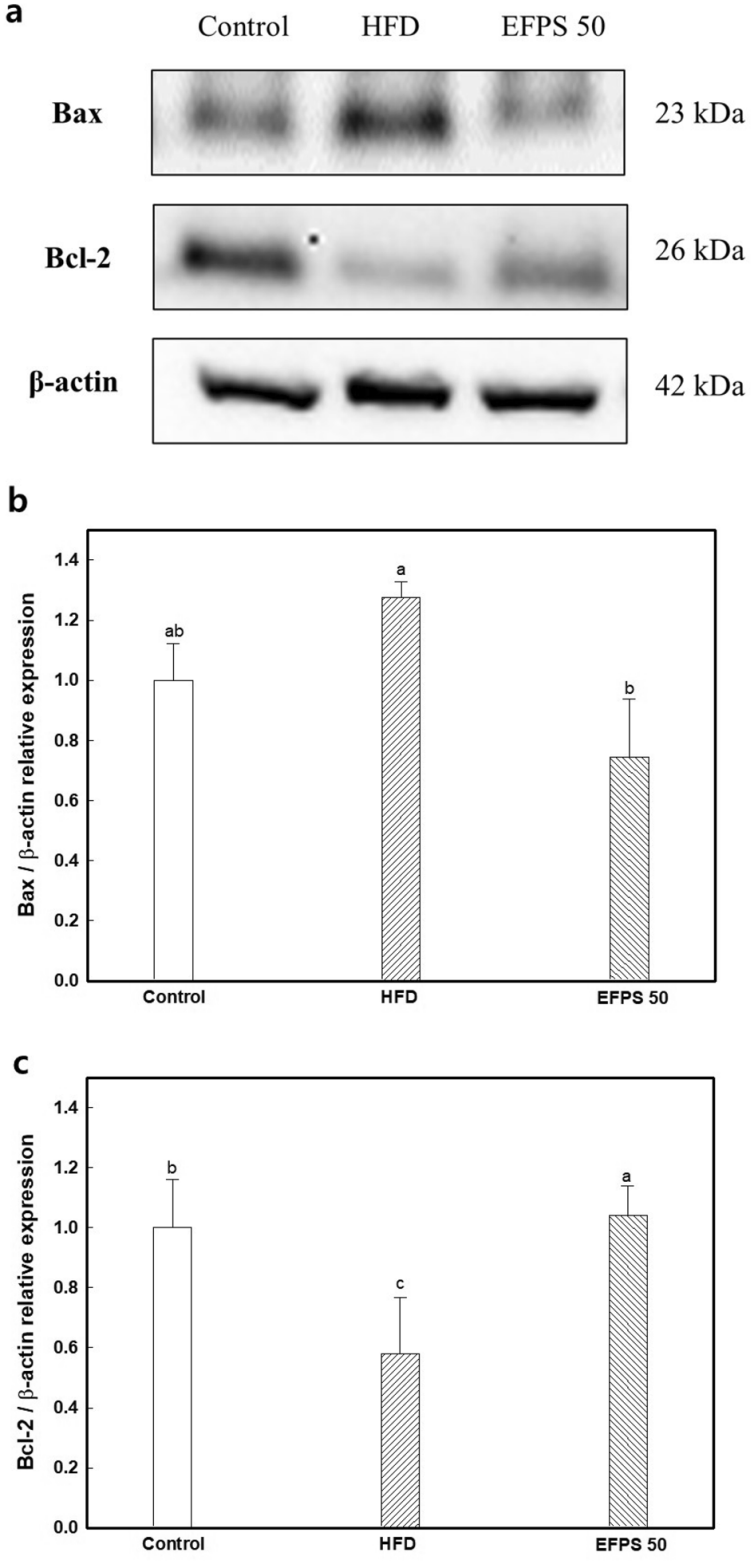
Effect of ethyl acetate fractions from peanut (*Arachis hypogaea*) sprout (EFPS) of neuronal apoptosis from brain homogenates of high fat-induced diabetic mice. Western blot band images (**a**), expression levels of Bax (**b**) and Bcl-2 (**c**). Results shown are means ± SD (n = 3). Data were statistically considered at *p* < 0.05, and different small letters represent statistical differences.

To confirm the effect of EFPS on apoptosis in brain tissue, the relative expression of proteins detected, and shown on Fig. 5. As the result of B-cell lymphoma 2 (Bcl-2), the expression level was decreased in the HFD group (0.58 ± 0.19) compared to the control group (1.00 ± 0.16). In addition, the HFD group showed increased expression level of Bcl-2 associated X (Bax) (1.27 ± 0.05). However, the EFPS 50 group significantly improved on Bcl-2 (1.04 ± 0.10) and Bax (0.74 ± 0.19) compared to the HFD group.

### Bioactive compounds analysis

**Figure 6:**
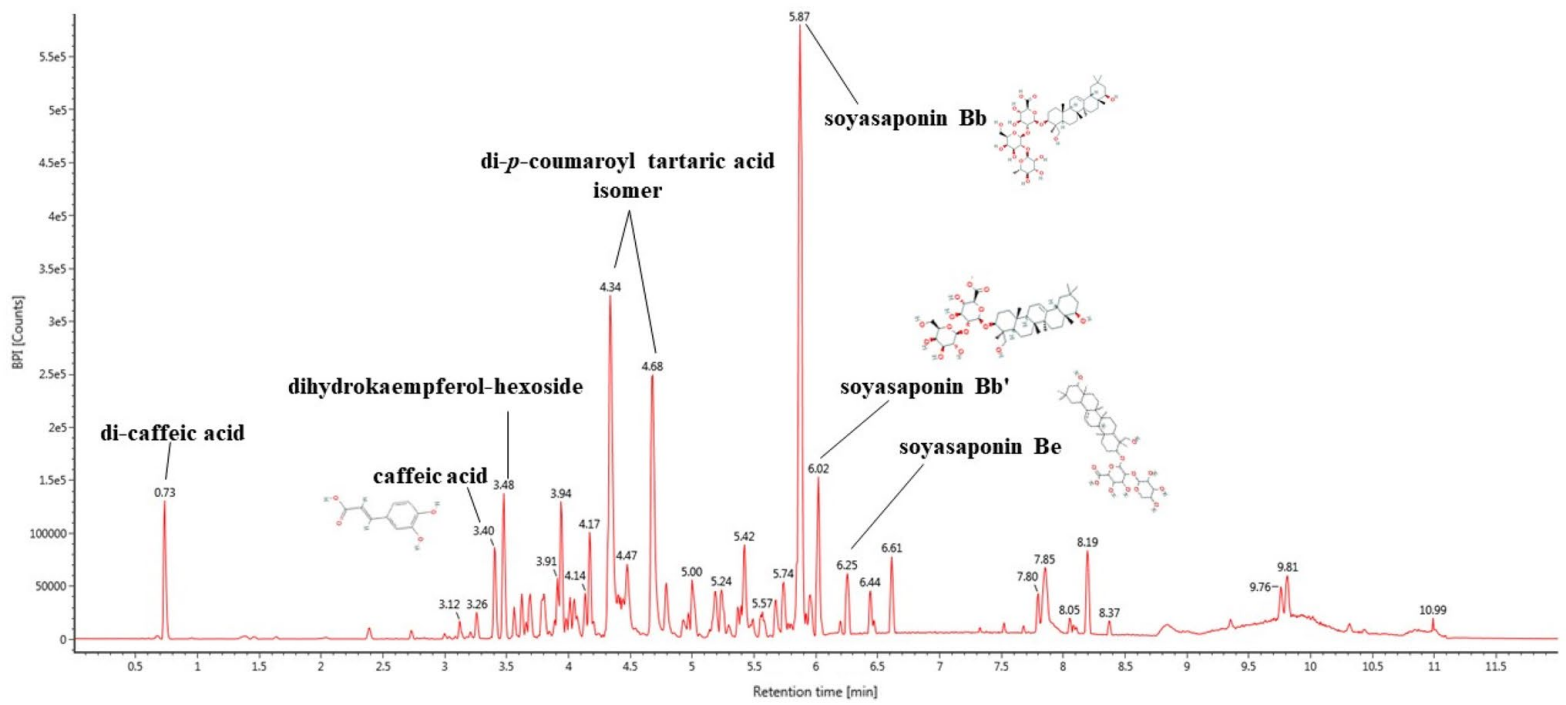
UPLC Q-TOF/MS^E^ chromatogram in negative ion mode of ethyl acetate fractions from peanut (*Arachis hypogaea*) sprout (EFPS).

The major bioactive compounds of EFPS were detected using a liquid chromatography quadrupole time-of-flight mass spectrometer (UPLC-Q-TOF/MS^E^) system. The main detected compounds were identified by comparing the LC/MS main fragments from previous literature and the Massbank database (Fig. 6, Table 4). A total of eight compounds were tentatively identified by MS fragmentations. Di-caffeic acid (RT: 0.73 min, m/z 341; 134, 135, 161, 179, and 683), caffeic acid (RT: 3.40 min, m/z 179; 134, 135 and 179) and dihydrokaempferol-hexoside (RT: 3.48 min, m/z 449; 287, 269, 259 and 243), di-*p*-coumaroyl tartaric acid isomer (RT: 4.34 and 4.68, m/z 441; 295, 277, 203, 163, 125 and 119), soyasaponin Bb (RT: 5.87, m/z: 941; 988), soyasaponin Bb′ (RT: 6.02, m/z: 795; 116, 788 and 925), soyasaponin Be (RT: 6.25, m/z: 939; 330, 342, 788, 985 and 1057), were identified as the main compounds in EFPS.

**Table 4:** Identification of main compounds in the ethyl acetate fraction from peanut (*Arachis hypogae*a) sprout (EFPS) by UPLC-Q-TOF/MS^E^ system.

Compound	Retention time (min)	Molecular formula	Precursor ion [M–H]^−^	Product ions
Di-caffeic acid	0.73	C_18_H_14_O_7_	341	134, 135, 161, 179, 683
Caffeic acid	3.40	C_9_H_8_O_4_	179	134, 135, 179
Dihydrokaempferol-hexoside	3.48	C_21_H_22_O_11_	449	287, 269, 259, 243
Di-*p*-coumaroyl tartaric acid isomer	4.34	C_22_H_18_O_10_	441	295, 277, 203, 163, 125, 119
Di-*p*-coumaroyl tartaric acid isomer	4.68	C_22_H_18_O_10_	441	295, 277, 203, 163
Soyasaponin Bb	5.87	C_48_H_78_O_18_	941	988
Soyasaponin Bb′	6.02	C_42_H_68_O_14_	795	116, 788, 925
Soyasaponin Be	6.25	C_48_H_76_O_18_	939	330, 342, 788, 985, 1057

## Discussion

Recent growing evidence demonstrates an association with obesity and cognitive impairment^15^. Chronic HFD intake induces obesity, T2DM, and impairment of short-term, working, and long-term memory^16^. In addition, the long-term consumption of HFD damages brain function via inflammatory response, insulin resistance, oxidative stress, and induction of mitochondrial dysfunction^17^. Therefore, the present study was conducted to assess the protective effect of EFPS on cognitive impairment caused by HFD.

Chronic HFD consumption increases free fatty acids decomposition, which can lead to an increase in very low-density lipoprotein (VLDL) and TG in blood^18^. In the hyperlipidemia state caused by HFD, insulin is involved in the production or removal of TG, causing metabolic disorders^19^. Insulin reduces hepatic gluconeogenesis and glycogenolysis and promotes TG storage in adipocytes, thereby reducing blood sugar level. However, chronic HFD intake induces insulin resistance in liver tissue, which directly interferes with glucose metabolism^20,21^. In this study, hyperglycemia and insulin resistance were induced in the HFD group (Table 1 and Fig. 1). However, EFPS restored insulin resistance by improving fasting blood glucose and impaired glucose tolerance. With these effects it is considered that EFPS improve insulin sensitivity and normalized blood glucose levels by reducing the accumulation of fat and suppressing abnormal blood lipid metabolism. In previous studies, group B soyasaponins were reported to improve the lipid profile by lowering TCHO, TG, LDLC and free fatty acids in serum^22^. In addition, they reduced hepatic TG accumulation by down-regulating the mRNA expression of sterol regulatory element-binding protein-1c (SREBP-1c) and fatty acid synthase (FAS) related to lipogenesis^23^. Considering these factors, group B soyasaponins might improve obesity-related metabolic dysfunction with hepatic insulin resistance in HFD mice. Therefore, it is considered that EFPS, which is rich in soyasaponin, might assist in the prevention of obesity and diabetes caused by HFD.

High glucose levels due to insulin resistance, which are derived early in T2DM, affect the brain^24^. In addition, high glucose levels damage the vulnerable brain from oxidative stress, causing impairment of learning and memory function in diabetic patients ^25,26^. Previous studies also proved that HFD could cause cognitive impairment^27,28^. In this study showed that HFD induced cognitive deficits in mice. However, EFPS improved learning and memory function impairment in the Y-maze, passive avoidance and Morris water maze behavioral tests (Fig. 2). Bioactive compounds such as caffeic acid, di-*p*-coumaroyl tartaric acid and group B soyasaponins were identified in EFPS (Table 4, Fig. 6). Caffeic acid improves memory impairment by ameliorating cerebral insulin and leptin signaling pathways in HFD-induced rats^29^. According to Hong et al.^30^, soyasaponin Bb protected learning and memory deficits by supporting neuronal regeneration processes and inhibiting hippocampal inflammation in cognitive impairment model rats. Moreover, it has been reported that soyasaponin Bb improves memory ability by increasing brain-derived neurotrophic factor (BDNF) protein expression in scopolamine-induced cognitive deficient mice^31^. In conclusion, it is considered that EFPS including various bioactive compounds considerably restored cognitive dysfunction.

The hyperglycemic state accelerates ROS production, lipid peroxidation oxidative modifications of DNA and proteins and in various tissues^32,33^. Especially, hepatic oxidative stress might interfere the insulin-signaling cascade and disturb with the normal transcription of certain proteins, induces insulin resistance^34^. Oxidative stress can cause liver cell damage and steatohepatitis by producing excessive oxidants and cytokines^35^. Also, the consequently increased ROS and reduced anti-oxidant activity, leading to damage on vascular cells, Schwann cells, neurons, as well as multiple other organs^36^. In particular, continuous hyperglycemia due to various causes such as obesity, promotes the production of ROS in brain tissue vulnerable to oxidative stress^37^. Brain tissue damaged by ROS and oxidative stress affects cognitive dysfunction in AD patients^38^. Based on previous studies, the antioxidant activities of liver and brain tissues were evaluated by measuring MDA content and SOD levels in this study. Consequently, EFPS reduced hepatic and brain oxidative stress caused by hyperglycemia (Fig. 3). *p*-Coumaric acid reduced HFD-induced oxidative stress by increasing antioxidant system in serum and liver tissues^39^. Moreover, it has been reported that consumption of caffeic acid improved the antioxidant system in the brain tissue of lipopolysaccharide (LPS)-induced neuroinflammation mice^40^. According to Salau et al*.*^41^, caffeic acid improved antioxidant enzymes by increasing glutathione, SOD, and catalase activities in brain tissues. These results suggest that EFPS can prevent damage to beta cells of the pancreas by protecting oxidative stress in the liver, and consequently contribute to the improvement of insulin resistance. EFPS is thought to protect liver tissue by activating the antioxidant system in the liver after damage induced by HFD. In addition, EFPS might improve cognitive dysfunction by protecting the antioxidant system in the brain against HFD-induced cytotoxicity.

The brain mitochondria play an important role by regulating calcium homeostasis, an important mechanism for energy demanding neurotransmission and learning and memory processes^42^. Also, the mitochondria contribute cellular energy generation in the form of ATP and maintain the balance between ROS production and detoxification^43^. However, excessive ROS production induces a variety of pathological conditions through oxidative damage in the brain^7,44^. Furthermore, when the formation of ROS is enhanced, it can impair mitochondrial function and the viability of cells^45^. Oxidative stress caused by high glucose damages mitochondria with rapid depolarization of MMP and generates more ROS through activation of inter-mitochondrial signaling networks^46^. Functional and structural of mitochondria degrades ATP production capability, which can lead to neurodegenerative diseases caused by mitochondrial disorders^6^. Previous studies have reported that consumption of HFD induces insulin resistance, which increases lipid peroxidation and oxidative stress. In addition, the excessive oxidative stress increases mitochondria membrane permeability, which promotes the secretion of apoptosis mediators such as Bcl-2 family and caspase^47^. Neuronal apoptosis is leads to neurodegeneration, ultimately leading to cognitive impairments such as AD. Therefore, many studies have been focused on finding agents that improve mitochondrial function, mainly based on the inhibition of oxidative stress. In the results of studying the protective effect of EFPS on mitochondrial deficit, EFPS significantly ameliorated mitochondrial dysfunction by restoration of mitochondrial ROS, MMT and ATP levels (Fig. 4).

A mitochondrial dysfunction such as decrease in MMP, increase of ROS, and depletion of ATP leads to apoptosis in neuronal cells^49^. Apoptosis of the intrinsic pathway is mostly inhibited or activated by Bcl-2 family proteins, and bcl-2 and bax proteins are known as representative cytotoxic mediators^48^. Cytosolic Bax combined with Bcl-2 in mitochondrial outer membrane and releases cytochrome c indicating caspase cascade by various stress such as DNA damage and ROS production. This process induces apoptosis and indicate the initiation of mitochondrial damage. In this study, the EFPS presented the regulation effect of protein expression related to apoptosis indicator (Fig. 5). The caffeic acids identified in EFPS reportedly have mitochondrial protective effects in relation to their antioxidant effects^50^. *p*-Coumaric acid upregulated the expression of nuclear respiratory factor 1 (NRF-1), a transcription factor that is a key mediator of genes involved in mitochondrial biosynthesis^51^. According to Ueda et al.^52^, *p*-coumaric acid improved mitochondrial dysfunction by controlling neuronal cell apoptosis caused by insoluble mutant copper-zinc SOD-1 aggregation on N2a cells (a mouse neuroblasts). It was also reported that p-coumaric acid protects neurodegeneration by regulation Bcl-2 and Bax proteins in hippocampal tissue in T2DM mice^53^. Based on these studies, it is considered that the mitochondrial-related apoptosis protein regulated effect of EFPS is due to the neuroprotective effect of various bioactive compounds contained in EFPS. These findings suggest that EFPS is considered a potential material for HFD-induced cognitive dysfunction by suppressing mitochondrial dysfunction.

In conclusion, this study showed that EFPS ameliorated serum lipid biomarkers and learning and memory function deficits by attenuating antioxidant system damage and brain mitochondrial dysfunction in HFD-induced diabetic mice. Thus, EFPS could be effective for a therapeutic strategy to improve neurodegenerative diseases such as AD caused by long-term HFD consumption. However, more studies are required to identify the detailed mechanism of EFPS on the role of mitochondrial function between HFD-induced insulin resistance and cognitive decline.

## Materials and methods

### Chemicals

Thiobarbituric acid (TBA), bovine serum albumin (BSA), ethylene glycol tetra-acetic acid (EGTA), 2-[4-(2-hydroxyethyl) piperazin-1-yl] ethane sulfonic acid (HEPES), 5,5,6,6-tetra-chloro-1,1,3,3-tetraethylbenzimidazolylcarbocyanine iodide (JC-1), 2′,7′-dichlorofluorescein diacetate (DCF-DA) and all other chemicals used were purchased from Sigma-Aldrich Chemical Co. (St. Louis, MO, USA). Superoxide dismutase (SOD) determination kit was purchased from Dojindo Molecular Technologies (Rockville, MD, USA). ATP assay kit was purchased from Promega Corp. (Madison, WI, USA). Primary antibodies for β-actin, Bcl-2 and Bax were purchased from Santa Cruz Biotechnology (Dallas, CA, USA). Secondary antibody for anti-mouse was purchased from Cell Signaling Technology (Danvers, MA, USA).

### Sample preparation

All methods were performed in accordance with the relevant guidelines and regulations. Peanut sprouts were supplied by Hansaeng Bio Co. Ltd. (Korea) in May 2017 and complied with relevant institutional, national, and international guidelines and legislation. The cultivation conditions of peanut sprouts are as follows. Peanuts cultivated in Gochang-gun (Korea) were verified from National Institute of Forest Science (Suwon, Korea). The obtained peanuts were dried in sunlight to lower the moisture content under 6–10%, and then dried peanuts were shelled. Peanut sprouts were obtained by repeating watering process at intervals of 5 h in a dark room at 26 °C for 7 days, and dried using hot air drying at 60 °C for 20 h (Lassele DY-220H, Busan, Korea). This incubated peanut sprouts were extracted in 50-fold 80% ethanol for 3 h at 40 °C. The extract was filtered and concentrated using a rotary evaporator (N–N series, Eyela Co., Tokyo, Japan). Then, the obtained concentrate was resuspended distilled water, and successively fractionated with n-hexane, chloroform and ethyl acetate. Through a preliminary test, the ethyl acetate fraction from peanut (*Arachis hypogaea*) sprout (EFPS) was used as an optimal extraction condition (Figs. S1 and S2). EFPS was lyophilized and powdered, and stored at -20 °C until use.

### Animals and experimental design

All animals were maintained and used in accordance with ARRIVE guidelines^54^ and all experimental procedures were approved according to guidelines established from the Institutional Animal Care and Use Committee of Gyeongsang National University (Certificate No. GNU-161116-M0065) on November 22, 2016. Male C57BL/6 mice (4 weeks old) were purchased from Samtako (Osan, Korea). Experiment condition was maintained under controlled temperature (22 ± 2 °C) and humidity (55%) with a 12 h light, 12 h dark cycles. The mice were randomly divided into 4 groups (n = 13; 8 for in vivo and ex vivo tests; 5 for mitochondrial tests), the control group was fed a normal diet, and the other groups were fed HFD for 15 weeks. To select the mice having hyperglycemia, the mice with a blood glucose level of 400 mg/mL or higher were selected, and randomly assigned to 3 mice per cage using the standard = RAND() function in Microsoft Excel. After, the EFPS dissolved in drinking water was intragastrically administrated to EFPS group (EFPS 20 and 50, 20 and 50 mg/kg of body weight, respectively) for 4 weeks. Control and HFD groups were provided by oral gavage the same dose of drinking water.

### Intraperitoneal Glucose Tolerance Test (IPGTT)

After mice were fasted overnight, d-glucose (2 g/kg of body weight) dissolved in 0.85% NaCl was intraperitoneally administered. Blood glucose was measured at 0, 15, 30, 60, 90 and 120 min using an Accu-Chek glucose meter (Roche Diagnostics, Basel, Switzerland) by taking blood from the tail of mice.

### Behavioral tests

#### Y-maze test

Mice were placed in a white construct consisting of a Y-maze with equal angles between three arms, and recorded for each mouse for 8 min. The movements of each mouse were recorded and evaluated using a video tracking system (SMART v3.0; Panlab, Barcelona, Spain)^55^.

#### Passive avoidance test

The passive avoidance apparatus made of lighted and darked chamber, separated by a central manual door. For the training trial, mice were initially placed in the lighted chamber for 1 min and then the lights were turned on for 2 min. The central separation door was opened, and when mice entered the dark chamber, electrical foot shock (0.5 mA) of 3 s was given to mice through the stainless rods. After 24 h, the latency time to re-enter the dark chamber was recorded for a maximum of 300 s^55^.

#### Morris water maze (MWM) test

MWM pool consist of a stainless steel circular pool randomly divided into four sections (E, W, S and N) of equal area. The pool was filled with water dissolved in white non-toxic ink, and water temperature was maintained 20 ± 2 °C. A white platform was placed in the W zone and submerged. The test was conducted for 6 days, and contained three parts of the visible platform test (day 1), the hidden platform test (day 2–5), and the probe test (day 6). The visible platform test was conducted with the platform visible on the water surface for 1 day. Hidden platform test was conducted for 4 days, when a mouse did not locate the platform within 60 s, it was placed to the platform and left for 20 s. In the probe test, the platform was removed and the retention time of each mouse in the W zone was recorded during 90 s^56^. All behavior tests were conducted in blinded condition with no information to investigators.

### Preparation serum and organs

After behavioral tests, the mice were sacrificed with CO_2_. Blood samples were collected from the postcaval vein and stored in heparin tube on ice. The brain, liver, spleen, kidney, white epididymal adipose and testis tissues were removed, rinsed with a saline and immediately weighed, and stored at − 70 °C. After the blood was centrifuges at 10,000*g* for 10 min at 4 °C, supernatant (i.e., serum) was immediately placed on ice and used for biochemical analysis.

### Serum biochemicals

The glutamic oxaloacetic transaminase (GOT), glutamine pyruvic transaminase (GPT), lactate dehydrogenase (LDH), blood urea nitrogen (BUN), creatine (CRE), total cholesterol (TCHO) and triglyceride (TG) were measured using clinical chemistry analyzer (Fuji dri-chem 4000i; Fuji film Co., Tokyo, Japan). Low-density lipoprotein cholesterol (LDLC) content and ratio of high-density lipoprotein cholesterol (HDLC) to TCHO (HTR) were calculated. HTR is calculated as follows: LDLC (mg/dL) = TCHO −  (HDLC + TG/5), HTR (%) = (HDLC/TCHO) × 100^57^.

### Antioxidant assay

#### Malondialdehyde (MDA) contents

To determine MDA contents, the brain and liver tissues were homogenized with phosphate-buffered saline (PBS) and centrifuged at 2500*g* for 10 min. The supernatants were reacted with 1% phosphoric acid and 0.67% TBA before they were incubated at 95 °C for 1 h. The absorbance was measured at 532 nm on microplate reader (Epoch 2, BioTek Instruments Inc., Winooski, VT, USA). The protein concentration was measured by the Bradford assay^58^.

#### SOD level

To determine SOD level, PBS was added of homogenized brain and liver tissues and centrifuged to obtain a pellet. Cell extraction buffer (10% SOD buffer, 0.4% Triton X-100, and 200 μM phenylmethane sulfonylfluoride) was added and then placed on ice for 30 min. Then, the extract was centrifuged at 10,000*g* for 10 min, and the supernatant was used for a SOD analysis. SOD level was determined according to the manufacturer’s protocol (Dojindo Molecular Technologies).

### Mitochondrial assay

#### Isolation of mitochondria

Brain mitochondria were isolated from whole brain tissue homogenized in the isolation buffer (1 mM EGTA, 0.1% BSA, 215 mM mannitol, 75 mM sucrose, 20 mM HEPES (Na^+^) and 75 mM sucrose, pH 7.2) and centrifuged at 1300*g* for 5 min. The supernatant was centrifuged at 13,000*g* for 10 min again. The pellet was mixed with the isolation buffer containing 0.1% digitonin and 1 mM EGTA, and incubated for 5 min. Then, centrifuged at 13,000*g* for 10 min. The obtained pellets were resuspended in isolation buffer and re-centrifuged. The final mitochondrial pellets were suspended in isolation buffer without EGTA and used for mitochondrial analysis^59^.

#### ROS production

The mitochondrial ROS production was measured using a respiration buffer (500 μM EGTA, 2 mM potassium phosphate, 20 mM HEPES, 125 mM KCl, 2.5 mM malate, 5 mM pyruvate and 1 mM MgCl, pH 7.0). A mitochondrial extraction was incubated with 25 μM DCF-DA in respiration buffer at room temperature. After 20 min, the fluorescence intensity was measured using fluorometer at excitation wave 530 nm and emission wave 590 nm (Infinite 200, Tecan Co., San Jose, CA, USA)^59^.

#### The mitochondrial membrane potential (MMP)

MMP was measured using the isolated mitochondria with a JC-1. The assay buffer contained mitochondrial isolation buffer with 5 mM malate and 5 mM pyruvate. The assay buffer and mitochondrial extraction were mixed on the 96-well black plate, addition of 1 μM JC-1. The fluorescence intensity was measured after 20 min (excitation; 530 nm and emission; 590 nm)^59^.

#### ATP contents

The ATP contents were determined on a luminescence meter (GloMax-Multi, Promega Corp.) using a kit (Promega Corp.) and calculated according a standard curve.

### Western blot assay

Brain tissues were homogenized with cold lysis buffer (ProtinEx Animal cell/tissue, GeneAll Biotechnology, Seoul, Korea) containing 1% protease inhibitor cocktails (Thermo Fisher Scientific, Rockford, IL, USA). And then, homogenates were centrifuged at 13,000*g* at 4 °C for 10 min. The supernatant was quantified by Bradford reagent (Bio-Rad, CA, USA) assay^58^. The samples were loaded on a 12% polyacrylamide gel and transferred onto a polyvinylidene fluoride (PVDF) membrane (Millipore, Billerica, MA, USA). After transfer, the membrane blocked by 5% skim milk, and reacted overnight in tris-buffered saline containing 0.1% Tween 20 (TBST) including each diluted primary antibody (dilution 1:1000) at 4 °C. Then, the membrane was washed twice with TBST and incubated with secondary antibody (dilution 1: 3000) for 1 h at room temperature. The washed membrane was detected with iBright CL1000 imager (Thermo Fisher Scientific, Waltham, MA, USA). The quantitative analysis of protein expression was performed by using Image J software (National Institutes of Health, Bethesda, MD, USA).

### Bioactive compounds analysis

The bioactive compound analysis of EFPS were analyzed using a UPLC-Q-TOF/MS^E^. A UPLC system (Vion, Waters Corp., Milford, MA, USA) was used and analytical samples were injected into an Acquity UPLC BEH C_18_ column (100 mm × 2.1 mm × 1.7 µm) (Waters Corp.). Gradient condition was conducted as 0.1% formic acid in distilled water (solvent A) and 0.1% formic acid in acetonitrile (solvent B) at a flow rate of 0.35 mL/min for 12 min. The gradient condition of mobile phase during analysis was applied as follows: 99% A/1% B at 0–1 min, 0% A/100% B at 7 min, 2% A/98% B at 8.5 min, 99% A/1% B at 10 min and 99% A/1% B at 12 min. The obtained data were analyzed using UNIFI software.

### Statistical analysis

All data were expressed as mean ± SD, and statistical analysis was analyzed by one-way analysis of variance (ANOVA) followed by Duncan’s multiple range test using SAS program (Ver. 9.4 SAS Institute, Cary, NC, USA). The statistical difference was presented as the different small letters (*p* < 0.05).

## Supplementary Information

Supplementary Information.
