# Supplementary material for: Peanut (Arachis hypogaea) sprout prevents high-fat diet-induced cognitive impairment by improving mitochondrial function
Source: Sci Rep. 2022 Apr 13;12:6213. doi: 10.1038/s41598-022-10520-5 (PMC9008020; doi:10.1038/s41598-022-10520-5)
Supplement: Supplementary file 1 — Supplementary Information. [file 41598_2022_10520_MOESM1_ESM.docx]

**Figure S1.** Effect of peanut (*Arachis hypogaea*) sprout fractions (a) and ethyl acetate fraction from peanut sprout (EFPS) (b) on lipid accumulation in 3T3-L1 adipocytes. Oil red O staining was used to visualize the lipid droplets to evaluate the differentiation of 3T3-L1 cells (c). Lipid droplets were stained in red. Data were statistically considered at 𝑝<0.05, and different small letters represent statistical difference.

**Figure S2.** Inhibitory effect of advanced glycation end products (AGEs) formation of ethyl acetate fraction from peanut (*Arachis hypogaea*) sprout (EFPS). Data were statistically considered at 𝑝<0.05, and different small letters represent statistical difference.

**Figure 5.** Effect of ethyl acetate fractions from peanut (*Arachis hypogaea*) sprout (EFPS) of neuronal apoptosis from brain homogenates of high fat-induced diabetic mice. Western blot band images (**a**), expression levels of Bax (**b**) and Bcl-2 (**c**). Results shown are means ± SD (n = 3). Data were statistically considered at *p* < 0.05, and different small letters represent statistical differences.


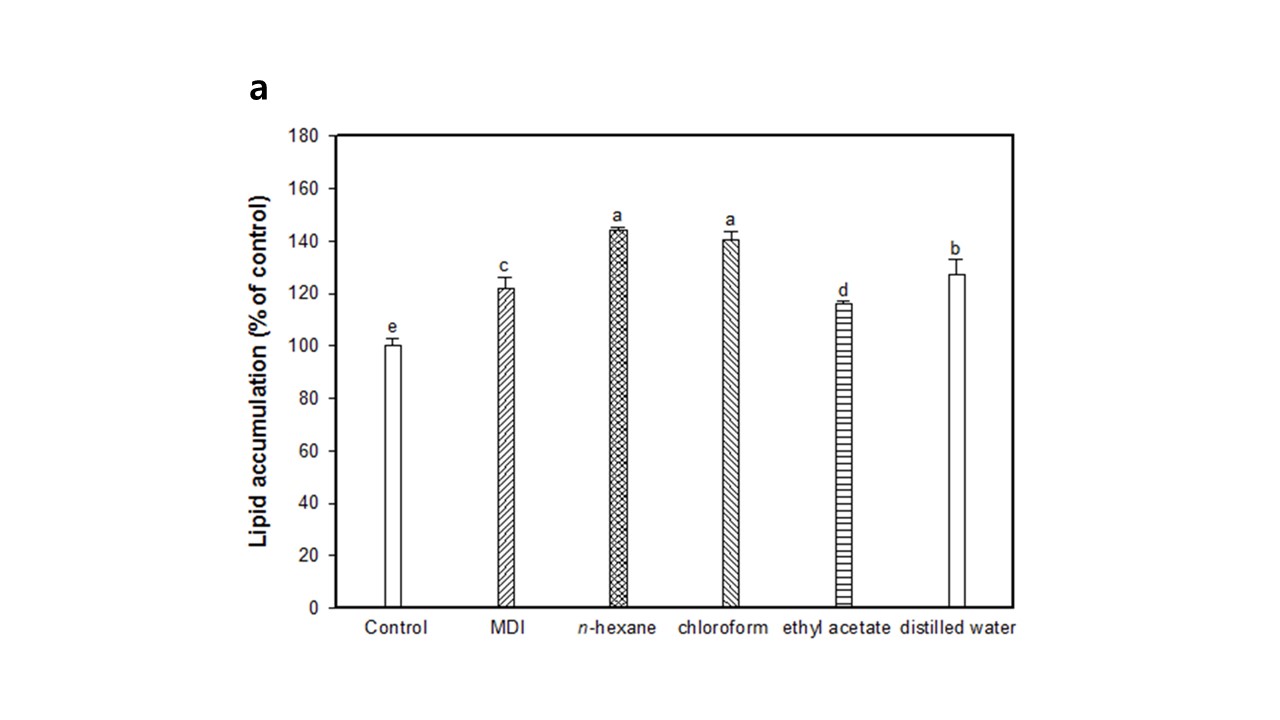


**
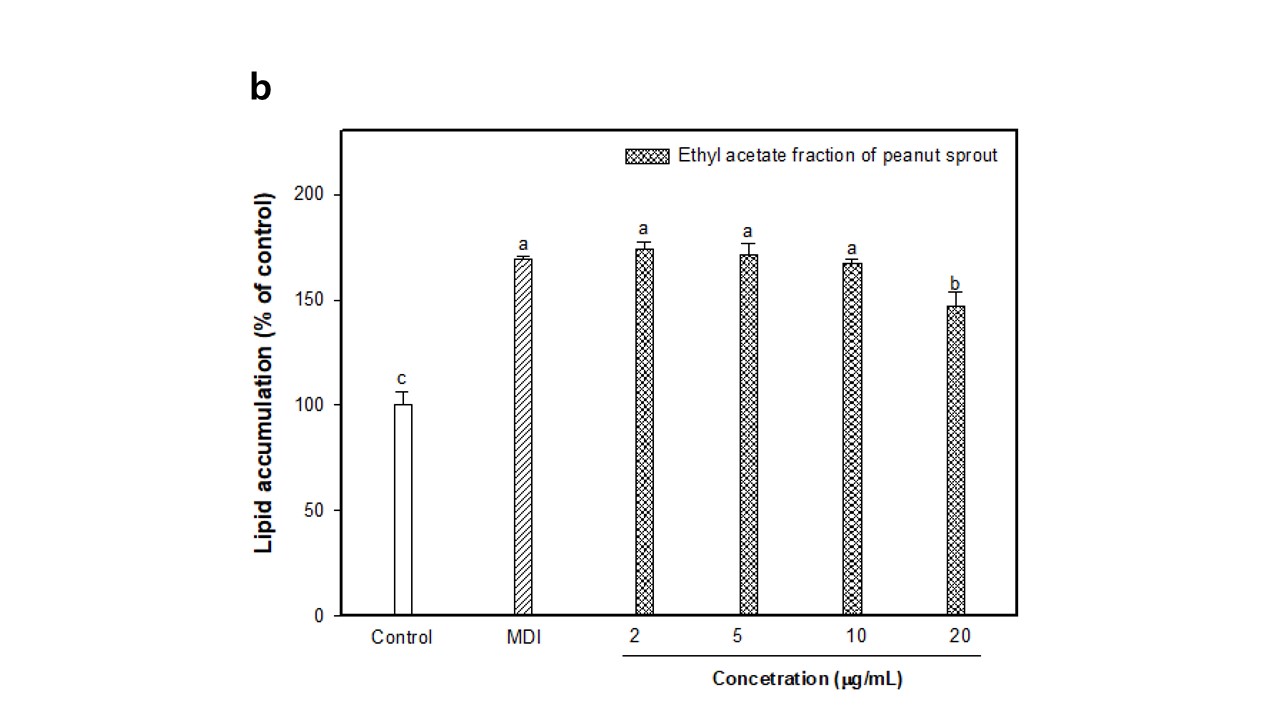
**

**
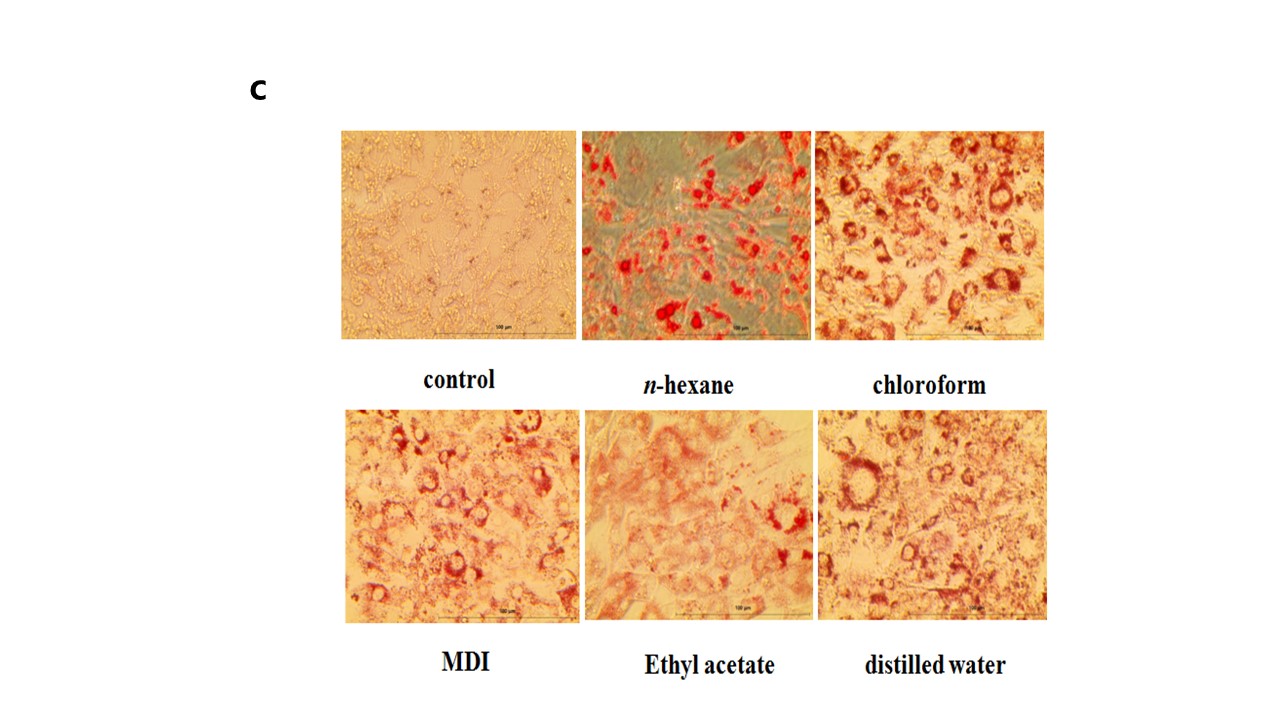
Fig. S1**


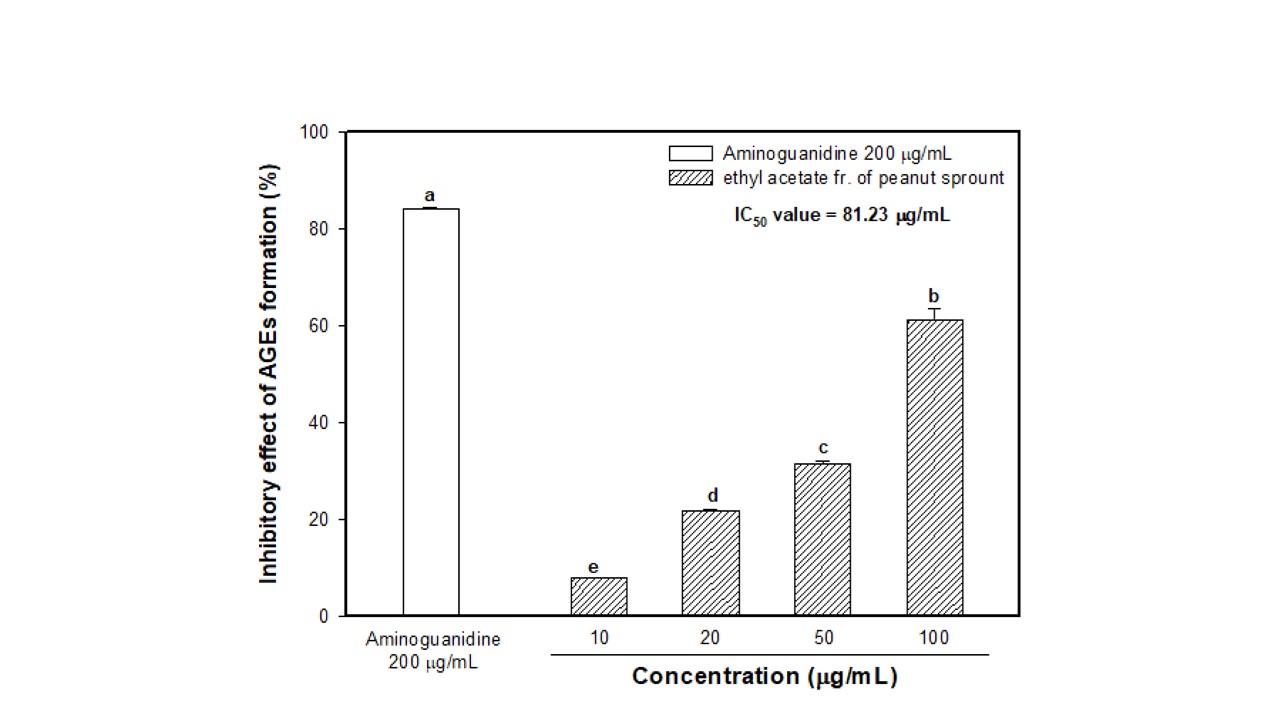


**Fig. S2**

**Bax**

**
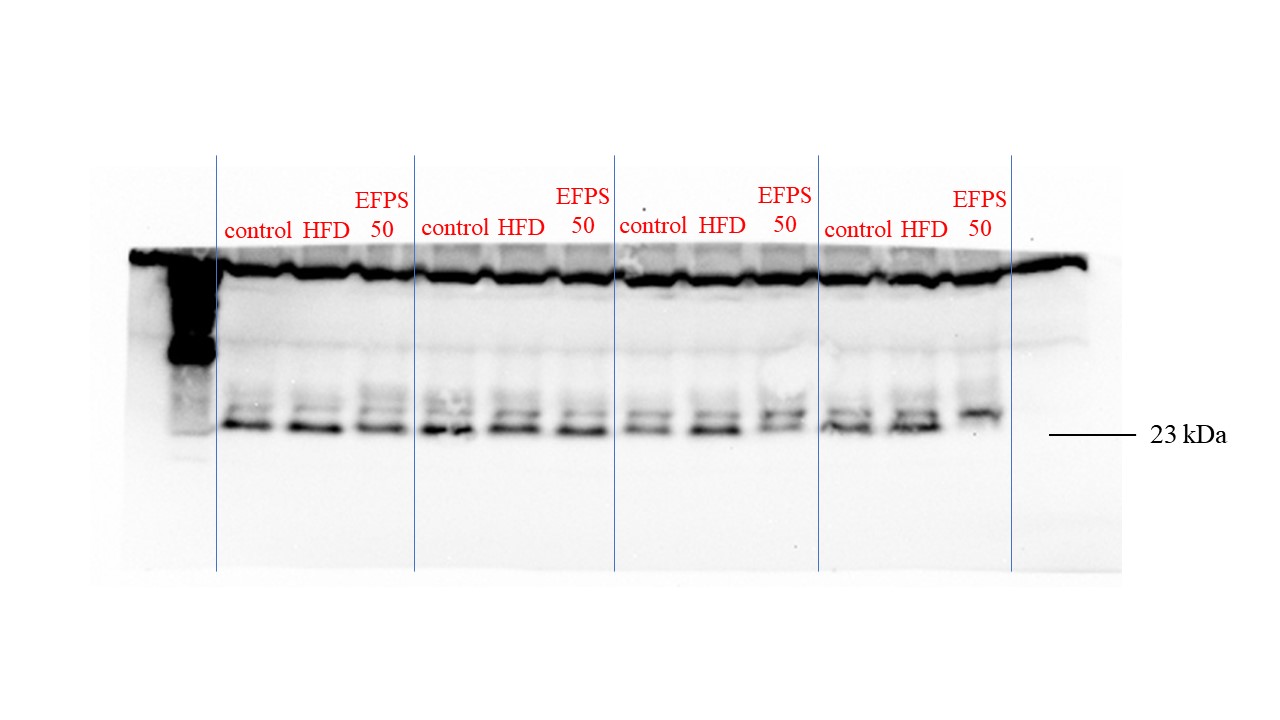
**

**Figure 5.** Effect of ethyl acetate fractions from peanut (*Arachis hypogaea*) sprout (EFPS) of neuronal apoptosis from brain homogenates of high fat-induced diabetic mice. Western blot band images (**a**), expression levels of Bax (**b**) and Bcl-2 (**c**). Results shown are means ± SD (n = 3). Data were statistically considered at *p* < 0.05, and different small letters represent statistical differences.


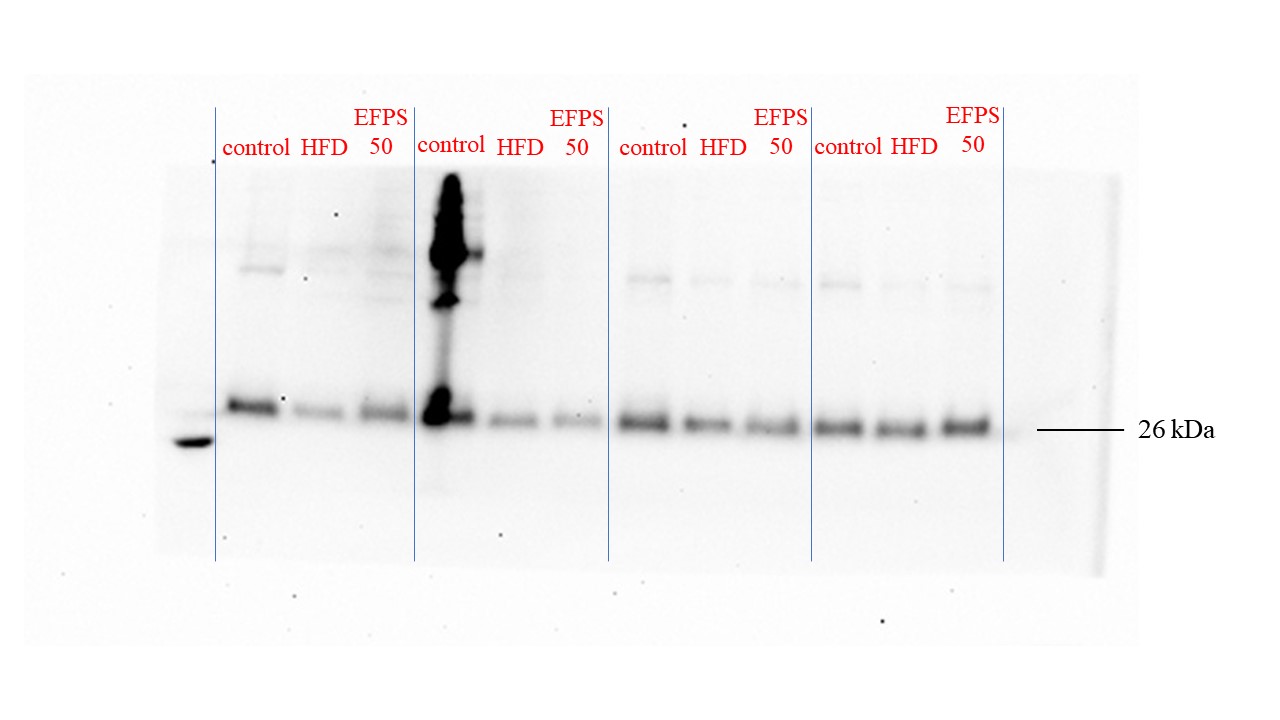
**Bcl-2**

**Figure 5.** Effect of ethyl acetate fractions from peanut (*Arachis hypogaea*) sprout (EFPS) of neuronal apoptosis from brain homogenates of high fat-induced diabetic mice. Western blot band images (**a**), expression levels of Bax (**b**) and Bcl-2 (**c**). Results shown are means ± SD (n = 3). Data were statistically considered at *p* < 0.05, and different small letters represent statistical differences.

**β-actin**


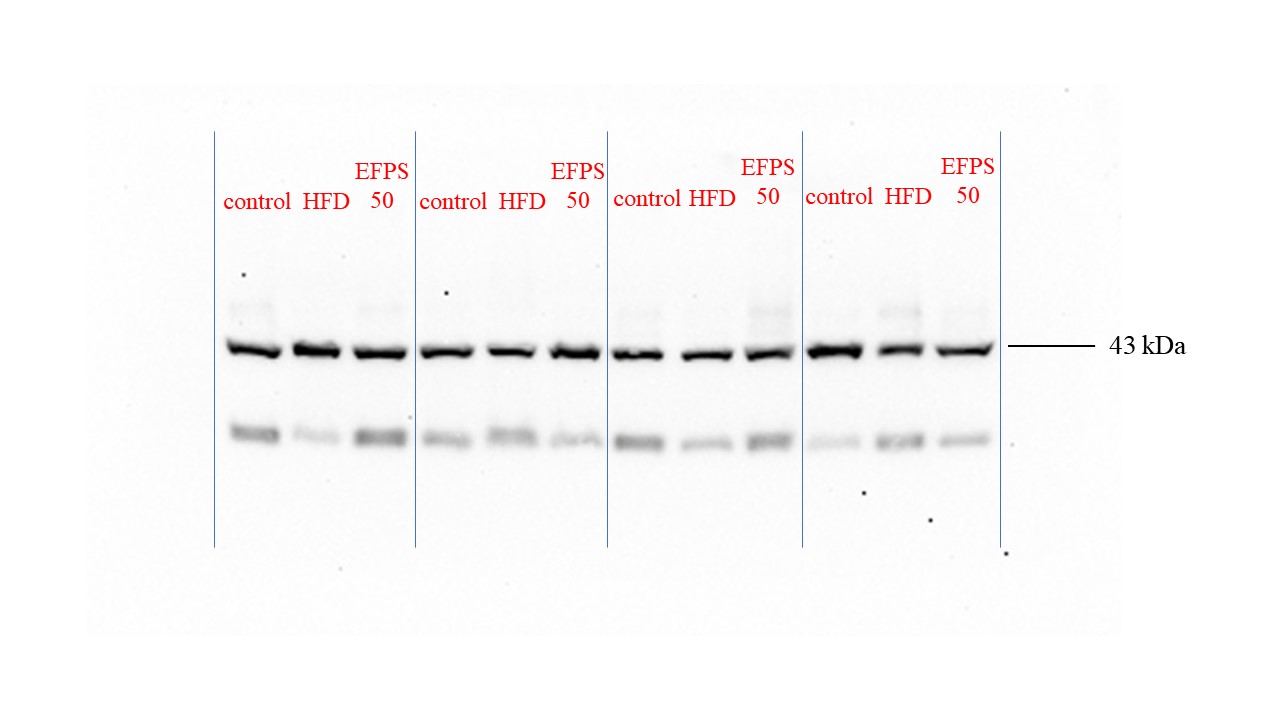
 **Figure 5.** Effect of ethyl acetate fractions from peanut (*Arachis hypogaea*) sprout (EFPS) of neuronal apoptosis from brain homogenates of high fat-induced diabetic mice. Western blot band images (**a**), expression levels of Bax (**b**) and Bcl-2 (**c**). Results shown are means ± SD (n = 3). Data were statistically considered at *p* < 0.05, and different small letters represent statistical differences.
